# Intensity-dependent gamma electrical stimulation regulates microglial activation, reduces beta-amyloid load, and facilitates memory in a mouse model of Alzheimer’s disease

**DOI:** 10.1186/s13578-023-01085-5

**Published:** 2023-07-28

**Authors:** Qian Liu, Adam Contreras, Muhammad Shan Afaq, Weijian Yang, Daniel K. Hsu, Michael Russell, Bruce Lyeth, Theodore P. Zanto, Min Zhao

**Affiliations:** 1 Institute for Regenerative Cures, Department of Ophthalmology & Vision Science, Department of Dermatology, University of California Davis, Sacramento, CA 95817 USA; 2 Department of Electrical and Computer Engineering, University of California, Davis, CA 95616 USA; 3 Department of Neurological Surgery, University of California, Davis, CA 95616 USA; 4 Neuroscape, Department of Neurology, University of California San Francisco, San Francisco, CA 94158 USA; 5 Institute of Biomedical and Health Engineering, Shenzhen Institutes of Advanced Technology (SIAT), Chinese Academy of Sciences, Shenzhen, China

**Keywords:** Alternating current stimulation, Gamma wave, Alzheimer’s disease, Microglia, Beta amyloid, Aβ, Learning and memory, 5xFAD mouse

## Abstract

**Background:**

Gamma sensory stimulation may reduce AD-specific pathology. Yet, the efficacy of alternating electrical current stimulation in animal models of AD is unknown, and prior research has not addressed intensity-dependent effects.

**Methods:**

The intensity-dependent effect of gamma electrical stimulation (GES) with a sinusoidal alternating current at 40 Hz on Aβ clearance and microglia modulation were assessed in 5xFAD mouse hippocampus and cortex, as well as the behavioral performance of the animals with the Morris Water Maze.

**Results:**

One hour of epidural GES delivered over a month significantly (1) reduced Aβ load in the AD brain, (2) increased microglia cell counts, decreased cell body size, increased length of cellular processes of the Iba1 + cells, and (3) improved behavioral performance (learning & memory). All these effects were most pronounced when a higher stimulation current was applied.

**Conclusion:**

The efficacy of GES on the reduction of AD pathology and the intensity-dependent feature provide guidance for the development of this promising therapeutic approach.

## Background

Alzheimer’s disease (AD) is a chronic and progressive neurodegenerative disorder causing 60–70% of cases of dementia. As the disease advances, there is a gradual loss of gray and white matter, deficient memory, and other cognitive dysfunction, and the disease ultimately leads to death [1, 2]. Among the aging population, AD represents one of the most significant, and ever-increasing morbidity in the US and around the world. Currently, there are 5.5 million Americans with AD and by the year 2050, the population of adults aged 65 + is expected to nearly double, and those afflicted with AD are expected to quadruple. The medical and related care costs in the US are estimated to be $236 billion, and projected to be more than $1 trillion by 2050 [3, 4]. In response to this public health crisis, extensive research has been aiming to remediate AD pathology.

A popular target for therapeutics has been beta-amyloid (Aβ), which is necessary, though not sufficient, for the pathogenesis of AD [5]. The amyloid cascade hypothesis of AD postulates that Aβ accumulation plays an important role in a chain reaction of events that leads to neuronal cell dysfunction and cell death, which gives rise to cognitive decline inherent to AD [6]. As such, numerous clinical trials have tested various pharmaceutical approaches toward remediating cognitive decline in AD by targeting Aβ load. Unfortunately, despite reductions in Aβ load, no pharmaceutical to date that targets Aβ, especially for the most toxic type for neuronal cells, Aβ42, has demonstrated efficacy in concurrently remediating cognition [7].

On the other hand, clinical electroencephalography (EEG) has revealed an association of AD with the increased power of low-frequency oscillations, and a decreased power of higher-frequency oscillations, i.e., gamma (30–60 Hz) [8, 9]. Research has suggested that gamma-band oscillations (30–100 Hz) play an important role as a primary stimulus among nerve cells and higher information processing in the brain [8]. A recent breakthrough has demonstrated that 40-Hz (gamma wave frequency) stimulation may offer a promising therapeutic approach to reduce AD-specific pathology and improve performance in behaviors in animal models of AD [10–14]. Interestingly, gamma stimulation of several different methods in animal models of AD has demonstrated efficacy, including optogenetic stimulation, visual/audiovisual sensory stimulation, or magnetic stimulation. Here, we attempt to extend this work to the electrical domain by applying sinusoidal alternating current stimulation within the gamma band (at 40 Hz). Moreover, previous assessments of gamma stimulation have not addressed potential intensity-specific effects. Therefore, this study will assess the intensity-specific efficacy of gamma electrical stimulation (GES), because proper intensity-based dosing is critical to the development of any therapeutic approach.

Electrical stimulation has been widely claimed to modulate brain function in humans [15, 16]. Most excitingly, GES in humans has recently been shown to significantly improve memory performances, along with restoration of intracortical connectivity measures of cholinergic neurotransmission, increased cortical blood perfusion, and possible reductions of tau [17–20]. Yet, the mechanisms of action rely on animal studies utilizing other forms of gamma stimulation. Here, we sought to fill this knowledge gap to address whether electrical stimulation similarly alters AD-specific pathology as other forms of gamma stimulation. Importantly, we will also assess fundamental unanswered questions regarding the intensity-specific (dose-response) effects of GES.

If GES has the same or similar effects on brain pathology and behavior performance in AD models, it will provide another powerful and practical modality to modulate gamma activities of the brain in patients. To test this, we established an epidural stimulation method guided by simulation of the electric field (EF) distribution within the brain. Here, we examined the effects of 40 Hz GES over a month in a 5xFAD mouse model of AD on Aβ loading, microglia morphology, and behavioral (memory) performance. Results demonstrated that biomarkers associated with AD pathology in both the hippocampus and cortex were significantly reduced and memory performance was significantly improved in an intensity-specific manner.

## Methods

### Animals and Alzheimer’s disease mouse model

This study was carried out following Animal Protocols #19,772 and #21,547 approved by the Institutional Animal Care and Use Committee (IACUC) at the University of California, Davis. The mice were housed in a temperature-controlled environment (22 ± 0.5 ℃) with a 12-hour-light-dark cycle and allowed free access to food and water. All efforts were made to ensure animal comfort and to reduce the number of animals used. We used 5xFAD mice as the Alzheimer’s disease model for the GES treatment in this study. The 5xFAD mice at the age of 3 months were divided into five groups: (1) sham, n = 6 mice; (2) 25 µA at 40 Hz (n = 8 mice); (3) 50 µA at 40 Hz (n = 8 mice); (4) 100 µA at 40 Hz (n = 6 mice); (5) 200 µA at 40 Hz (n = 7 mice). The 5xFAD transgenic mice (B6.Cg-Tg (APPSwFlLon, PSEN1*M146L* L286V) 6799Vas/Mmjax) were purchased from the Jackson Laboratory (RRID: MMRRC_034848-JAX). As described previously [21], the 5xFAD strain overexpresses both mutant human amyloid beta (A4) precursor protein 695 (APP) with the Swedish (K670N, M671L), Florida (I716V), and London (V717I) Familial Alzheimer’s Disease (FAD) mutations and human presenilin 1 (PS1) harboring two FAD mutations, M146L and L286V. 5xFAD transgenic mice recapitulate major features of Alzheimer’s Disease amyloid pathology and may be useful models of intraneuronal Aβ42 induced neurodegeneration and amyloid plaque formation. 5xFAD mice generate Aβ42 almost exclusively, rapidly accumulating high cerebral levels. On this mixed C57BL/6 and SJL background (MMRRC stock 34,840), intraneuronal Aβ42 accumulation is observed starting at 1.5 months of age, just before amyloid deposition, and gliosis, which begins at two months of age.

### Electrode implanting surgery

**Fig. 1:**
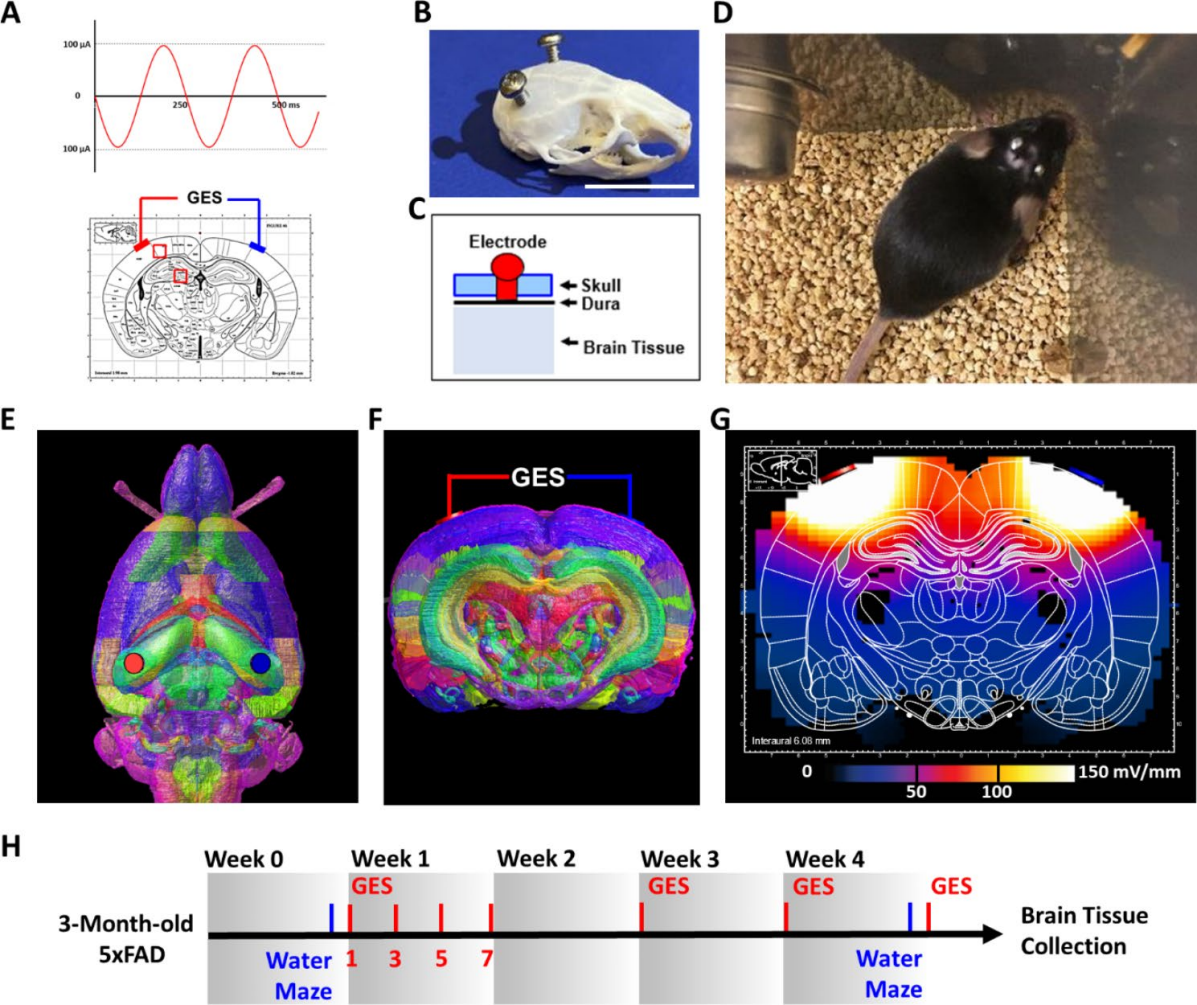
**Intracranial GES for 5xFAD mouse and the FEM simulation analysis** **A-C.** Electrode implantation. Two stainless steel screws were implanted as the paired electrodes to deliver the intracranial sinusoidal alternating current stimulation (A), as shown in the skull at: Anterior-Posterior (AP) = -2 mm, and Medial-Lateral (ML) = 4 mm (left and right) to the bregma (B-C). The electrodes were screwed and immobilized in the skull with the distal end reaching the dura. The positioning of the electrodes was determined following computer simulation that indicates maximal electric fields/currents to the targeted regions – cortex and hippocampus [15] **D.** A mouse with electrodes implanted. Following the implantation surgery, the animals were checked twice daily to ensure no infection, no changes in health and behaviors, and normal activities until the end of the experiment **E-F.** Three-dimensional (3D) simulation of the distribution of electric fields in a mouse brain, based on C57BL/6 mouse brain atlas with MRI and Nissl histology, with 39 regions represented (in different colors). The simulation helped to determine the positioning of the electrodes (in red and blue) to achieve desired field distribution as in G **G.** The Finite element method (FEM) simulation suggests electric field (EF) distribution in the brain, which would effectively stimulate the cortex (with EF intensity at ~ 100–150 mV/mm) and hippocampus (with EF intensity at ~ 10–80 mV/mm), two main regions affected by Aβ overload in AD. **H.** Behavior tests (blue bars) and Gamma Electric Stimulation (GES, red bars). The 5xFAD mice (3-month-old, male) were randomly assigned into sham, Low-current, and High-current groups. Before the GES, the Morris Water Maze assessment consisted of training and probe trials performed in Week 0 (blue bar). The electrodes were implanted 24 h before the first stimulation. The stimulation was delivered (red bars) for 1 h each day on Monday, Wednesday, Friday, and Sunday of Week 1; for 1 h on Monday of Week 3 and 4, and then for 1 h on Sunday of Week 4, followed by immediate euthanization and brain tissue collection. The GES details were: 40 Hz at 25 µA (n = 8), 50 µA (n = 8), 100 µA (n = 6), and 200 µA (n = 7) (amplitudes produced and monitored by the Neuroelectrics® Starstim®). The sham mice (n = 6) underwent every procedure, except that GES was not switched on. The Morris Water Maze test was performed in Week 4 (blue bar) as the learning and memory behavior assessment after the 4-week GES respectively

The electrode implanting surgery was performed on all 5xFAD mice from each of the five experimental groups 24 h before the first GES session. As described previously [21], the mice received anesthesia with 2% (v/v) isoflurane in oxygen (0.2–0.5 L/min) before surgery. The animal was then mounted on a stereotactic apparatus and received a small (5–8 mm) scalp incision followed by two burr holes with a diameter of 1.5 mm, using a dental drill, at the coordinates: Anterior-Posterior (AP) = -2 mm, and Medial-Lateral (ML) = 4 mm left for one electrode and 4 mm right for the other electrode, relative to the bregma. Two stainless steel screws (0–80, DIA: 0.06 inch) were sterilized and implanted into the burr holes as electrodes (Fig. 1A-D). The electrodes were implanted to a depth of 0.5–0.8 mm from the bone surface, only touching, but not penetrating the dura (Fig. 1C). After the implantation, the electrodes were fixed with dental cement. During the entire surgery, the mouse was placed on a thermostatically controlled warming pad, and body temperature was monitored with a rectal thermometer. The depth of anesthesia was monitored every 10 min by a toe pinch to elicit a foot withdrawal. For the analgesic regimen, the mice received subcutaneous Carprofen at 2 mg/kg at the time of surgery. The mice were assessed twice daily in the following two days after the surgery, and Carprofen (2 mg/kg) was administered if the mice showed signs of pain or stress.

### Mouse brain modeling and the FEM simulation

To assess the coverage and strength of GES to brain regions within the 5xFAD mice, such as the cortex and hippocampus, we applied the finite element method (FEM) to estimate the derived electric field (EF) distribution and intensity in a three-dimensional mouse brain model (Fig. 1E and G). We built the mouse brain model as reported previously [21], based on a 3D C57BL/6 mouse brain atlas built from MRI and Nissl histology, which consists of 39 different brain regions (Fig. 1E F) [22]. The regions were grouped as dura, arachnoid, grey matter, white matter, or cerebral ventricles, and assigned the relative electrical conductivity and relative permittivity (at 40 Hz, stimulation frequency used in the study) [23]. The defined 3D model was then rendered, so it contains a total of 107 × 152 × 105 voxels with voxel resolutions ~ 100 × 100 × 100 µm^3^. Modeled electrodes were placed over the dura through a craniotomy hole (Fig. 1E F, in red and blue). We used the Sim4Life platform (v4.4.2.3851, Zurich MedTech AG) to perform a quasi-electrostatic FEM simulation to calculate the derived EF distribution and intensity in the brain model (Fig. 1G).

### Gamma electrical stimulation

GES was administrated through the implanted electrodes 24 h after the surgery. Before GES treatment, the 5xFAD mice were anesthetized with ketamine/xylazine (90/4.5 mg/kg, i.p.). The GES stimulating program was set as 40 Hz at 25 µA (n = 8 mice), 50 µA (n = 8 mice), 100 µA (n = 6 mice), and 200 µA (n = 7 mice). Stimulation was applied 1 h/day, on every other day of the 1st week; no stimulation in the 2nd week; for 1 h on Monday of the 3rd week; 1 h/day, on Monday and Sunday in the 4th week (Fig. 1H). For the Sham group (n = 6), the mice received electrode implantation and anesthesia during the 4-week treatment duration, but with no GES. For the 4-week GES treatment, the body weight and neurological behavior were monitored once each day to assess the safety of GES on 5xFAD, as described previously [21]. After the programmed 4-week GES treatment, the mice were euthanized with overdosed CO_2_. Cardiac perfusion with ice-cold 0.1 M phosphate buffer (PB) solution was performed to collect the brain tissue. Both hemispheres of the collected sham and GES-treated 5xFAD mouse brains were then separated in ice-cold 0.1 M PB solution. The left hemispheres were used for ELISA, and the right hemispheres were fixed in ice-cold 4% paraformaldehyde (PFA) for the following immunofluorescence analyses.

### Enzyme-linked immunosorbent assay (ELISA)

The collected left hemispheres of 5xFAD mouse brains were dissected within an ice-cold 0.1 M PB solution. The hippocampus and cortex were separately collected in pyrogen/endotoxin-free tubes, for ELISA of Aβ42 and Aβ40. When analyzing samples, ~ 100 mg of the hippocampus or cortex was weighted. The tissue was then added into 800 µL 5 M guanidine-HCl (G4505-100G, Sigma-Aldrich, MO, USA)/50 mM Tris (T1378, Sigma-Aldrich, MO, USA) solution, pH 8.0, containing the Protease Inhibitor Cocktail and AEBSF (P2714-1BTL, Sigma-Aldrich, MO, USA). The mixture was then respectively homogenized at room temperature for 3.5 h. The homogenate was then diluted with a ten-fold volume ice-cold 0.1 M PB solution containing protease inhibitor cocktail, and centrifuged at 16,000 g for 20 min, at 4 °C. The supernatant was then harvested for ELISA of Aβ42/Aβ40 using the Mouse Aβ42 ELISA Kit (KMB3441-96 tests, Invitrogen) or the Mouse Aβ40 ELISA Kit (KMB3481-96 tests, Invitrogen), following the manufacturer’s instruction. After the anti-Aβ42, IgG HRP, and chromogen incubations, the 96-well plate was placed in a Microplate Spectrophotometer (BioTek, US) to read the absorbance at 450 nm. The concentrations of the samples were read from the stand curve, generated according to the Aβ42 standard.

### Immunofluorescence

The collected right hemispheres of 5xFAD mouse brains were used for immunofluorescence of NeuN/Aβ42, NeuN/Aβ40, and Iba1/Aβ42. The hemisphere samples were fixed in ice-cold 4% PFA at 4 °C for 3 days, and transferred into a 30% (v/v) sucrose solution at 4 °C for tissue dehydration for 3 days. Afterward, the hemispheres were respectively frozen and coronally sectioned at 40 μm intervals with a cryostat microtome. To detect protein expressions in the hippocampus and cortex, specific hemisphere sections were collected following the previous description [21, 24, 25]: coronal sections between AP: -1.2 mm and − 2.7 mm (1.5 mm in thickness) from the bregma.

The 40 μm hemisphere slices were then further fixed in 4% PFA for 30 min and permeabilized in 0.1% Triton X-100 (T8787, Sigma-Aldrich, MO, USA) for 30 min. The slices were then incubated in 3% bovine serum album (BSA) in 0.1 M phosphate-buffered saline (PBS) solution at room temperature for 1 h. After the non-specific protein blocking, the slices were incubated with diluted primary antibodies: anti-NeuN (1:1000, MAB377, Sigma-Aldrich, MO, USA), anti-Aβ42 (1:1000, #805,501, BioLegend), anti-Aβ 40 (1:1000, #805,401, BioLegend) and anti-Iba1 (1:1000, #019-19741, FUJIFILM Wako Chemicals USA) at 4° C, overnight. The slices were then rinsed with 0.1 M PB solution three times at room temperature and incubated with diluted goat anti-mouse/rabbit (Alex Fluor 594/488, 1:1000, #A-11,005, #A-11,034, Invitrogen) secondary antibodies. ProLong Gold Antifade Mountant with DAPI (P36931, Invitrogen) was used to label nuclei and fix the final slice for fluorescence detection with confocal laser-scanning microscopy (Leica SP8 STED 3X microscope with 20X and 63 × 1.4 NA objectives).

### Quantification of immunopositive cells

As previously published [21], the Iba1 immunopositive cell counting, cell body diameter, and process measurements in the hippocampus and cortex were performed with ImageJ software. Specifically, we used a stereological method to obtain coronal brain sections at 40 μm intervals for immunofluorescence. We used 20X and 10X objectives to identify Iba1 + cells at 5 section intervals (200 μm apart), and within each section, we selected 3 fields of view from an area of 200 × 200 μm² for cell counting. We calculated cell counts and measurements for the hippocampal and cortical regions in each animal and then averaged them to obtain group means and standard errors of the mean (SEM), following the systematic random sampling protocol of stereology [26, 27]. We have used this method in our previous research publications, particularly in studies involving the DG region of the hippocampus [21, 24, 25]. The DG region of a mouse can be easily detected in almost full view under the 20X and 10X confocal microscope in three snapshots in the coronal plane. Therefore, the DG region was not manually selected, but captured in its entirety. In the sagittal plane, we collected data from almost all sections to ensure that we did not miss any stereological cell count data. The cell counts and measurements in the hippocampal and cortex regions of each animal were calculated and averaged to obtain the group means and standard error of the mean (SEM).

### The morris water maze (MWM)

The MWM consisted of a 4-day training session and a 60-second assessment session on Day 5. For each training session, mice were placed into the pool at one of four semi-randomly chosen starting points and given 60 s to reach the platform. Any mouse that does not reach the platform within 60 s was led to the platform by the experimenter and allowed to remain on the platform for 10 s. Twenty-four hours after the final training session, mice were given an assessment session lasting 60 s, where the platform had been removed from the pool. Swim paths were recorded using a video tracking system. During training sessions, swim distance, latency to reach the platform, and swim speed were measured. During the assessment session, swim distance, swim speed, swim time in each quadrant, and the time spent in the platform zone were measured. Efficiency was calculated as the ratio of the direct distance from the start point to the hidden escape platform to the actual distance the mouse swam from the start point to the platform. To assess episodic memory, assessment data (day 6) was submitted to an ANOVA with Group (Sham, Low-current, high-current) and Session (pre-GES, post-GES) as factors. To assess learning, data from the training sessions were modeled across days, and slopes of best fit were submitted to the same ANOVA as before.

### Statistics

Data analysis was performed using GraphPad Prism 8 (GraphPad Software, Inc., CA, US), which adheres to a general linear model. The alpha level for Type I error was set at 0.05 for rejecting null hypotheses. Data were expressed as mean ± standard error of the mean (SEM). Microglia cell activation assessed from Iba1 staining was separately analyzed by one-way ANOVA for each group, followed by a Tukey’s Honestly Significant Difference *post-hoc* analysis for the 5xFAD + sham and 5xFAD + GES group comparisons.

## Results

### GES decreased Aβ42 and Aβ40 load in the hippocampus and cortex of 5xFAD mice

**Fig. 2:**
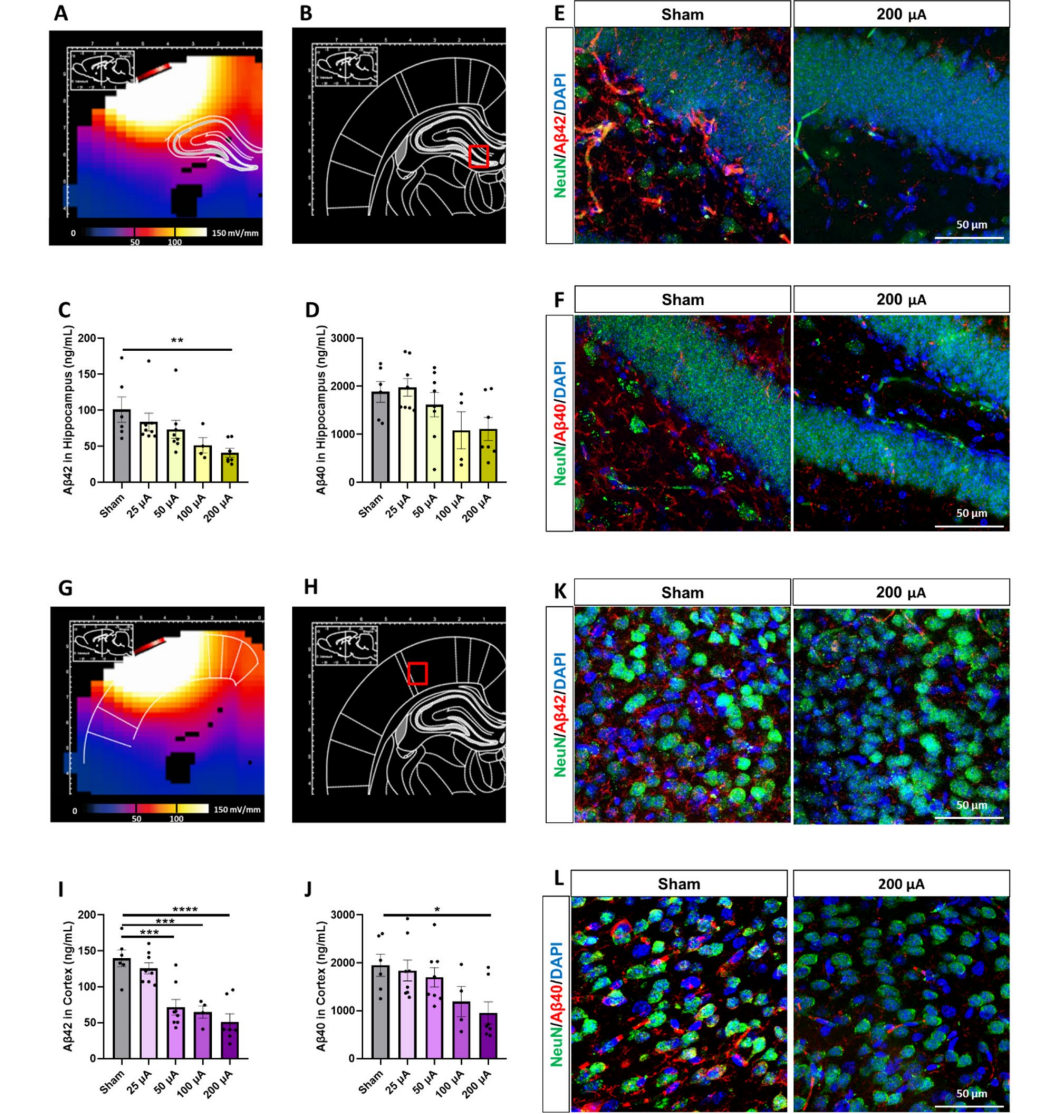
**GES decreased Aβ42 and Aβ40 in the hippocampus and cortex of 5xFAD mice** **A, G.** EF distribution to the hippocampus (**A**) and cortex (**G**) simulated by the FEM. **B, H.** Following the 4-week GES, the hippocampus (**B**, red box) and cortex tissues (**H**, red box) of 5xFAD mice from each group were separately collected for Aβ42/ Aβ40 ELISA assay and immunofluorescence microscopy. **C-D, I-J.** ELISA assay showed that the GES treatment significantly decreased Aβ42 and Aβ40 concentrations in the hippocampus (**C-D**,) and the cortex (**I-J**) following GES in 25, 50, 100, and 200 µA groups. **E-F, K-L.** Typical example immunofluorescence images show significantly decreased Aβ42 (red in **E** and **K**) and Aβ40 (red in **F** and **L**) labeling in the dentate gyrus (DG) of the hippocampus (**E-F**) and in the cortex (**K-L**) following 200 µA GES. NeuN (green) and DAPI (blue) were used to label neurons and nuclei. Scale bars: 50 μm Data were presented as mean ± SEM. * P < 0.05, ** P < 0.01, and *** P < 0.001 were considered as significantly different for GES groups vs. sham

The FEM simulation suggested that GES delivered from the electrodes as positioned effectively reached the cortex and the hippocampus. For example, GES of 40 Hz at 100 µA induced 10–80 mV/mm in the hippocampus (Fig. 2A) and ~ 100–150 mV/mm in the cortex (Fig. 2G). These regions were then assessed in the following analyses utilizing ELISA and immunofluorescence microscopy.

After the 4-week stimulation program (Fig. 1H), brain tissue was collected for Aβ42 and Aβ40 load detection (Fig. 2B H). Positioning of the electrodes was selected for effective delivery of electric stimulation to the cortex and the hippocampus of the mouse brain as previously simulated and detailed [21]. The Aβ42 and Aβ40 concentrations from these two regions were subsequently quantified via an ELISA immunoassay and assessed with a one-way ANOVA to compare the GES groups and Sham. Results of the ANOVA exhibited a main effect (Aβ42: F(4, 28) = 3.440, P = 0.0208, for the hippocampus; F(4, 28) = 14.32, P < 0.0001, for cortex; Aβ40: F(4, 28) = 2.748, P = 0.0480, for the hippocampus; F(4, 28) = 3.386, P = 0.0222 for the cortex). The Aβ42 concentration in sham 5xFAD mice was quantified as 101.0 ± 17.6 ng/mL in the hippocampus and 139.7 ± 11.5 ng/mL in the cortex (Sham). Compared to the Sham group, the Aβ42 concentration in the 200 µA GES group was significantly decreased to 41.2 ± 6.1 ng/mL (P = 0.0089) in the hippocampus and 51.3 ± 11.1 ng/mL (P < 0.0001) in the cortex. On the other hand, compared to the Sham group, the Aβ40 concentration in the 200 µA GES group showed no significant change in the hippocampus, but was significantly attenuated to 956.2 ± 230.0 ng/mL (1950.0 ± 230.4 in sham, P < 0.0001) in the cortex (Fig. 2C, D and I J). To confirm the ELISA result, the Aβ42 and Aβ40 immunofluorescence was also performed. Visual inspection and quantification of the immunofluorescence data indicated that the sham 5xFAD brains loaded relatively more Aβ42 and Aβ40 in the hippocampus and cortex, while the Aβ42 and Aβ40 load was decreased in the 200 µA GES treatment groups. (Figure 2E F and 2 K-2 L).

### GES-regulated microglial activation in the hippocampus of 5xFAD mice

**Fig. 3:**
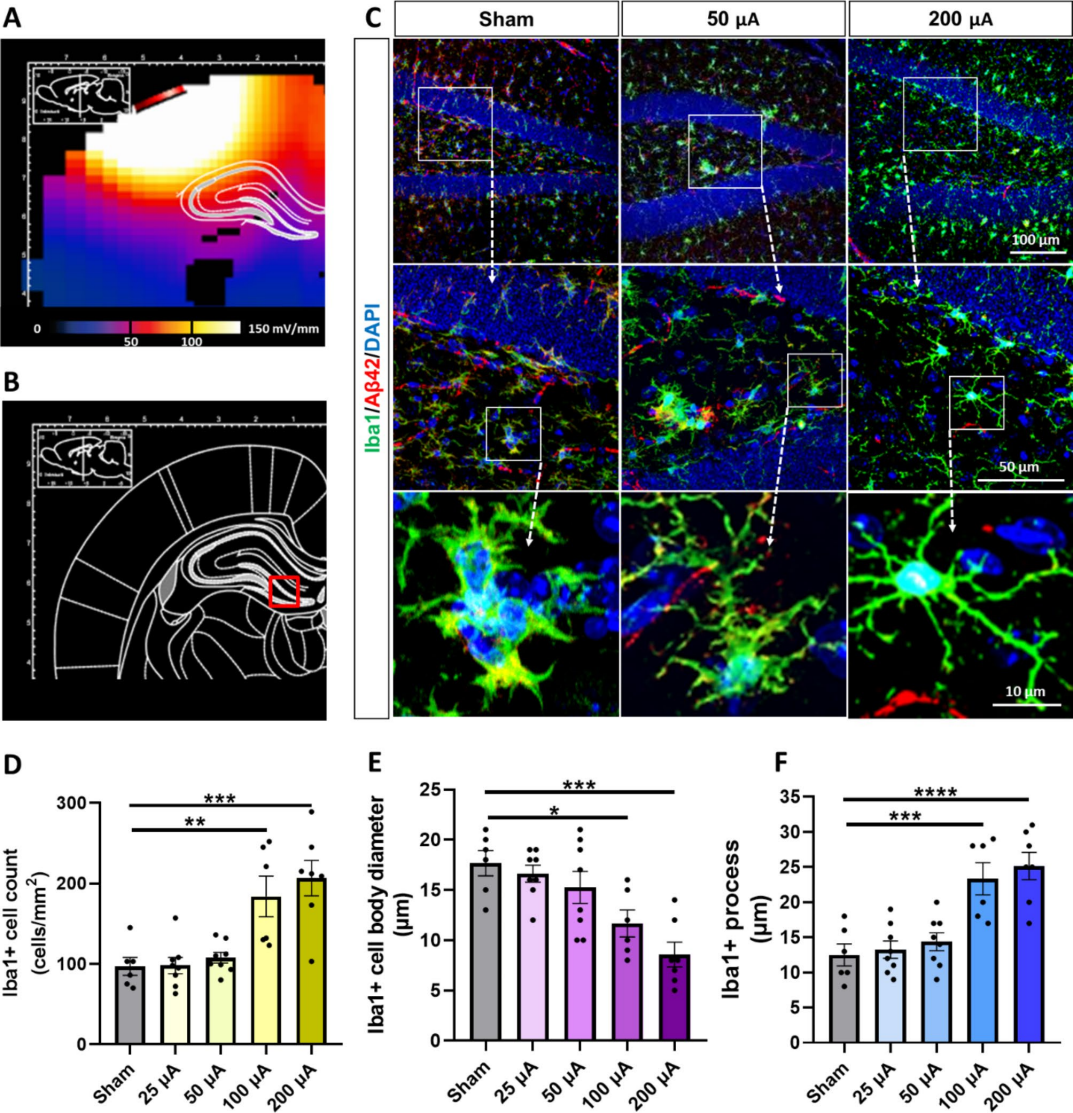
**The GES modulated microglia activation in the hippocampus of 5xFAD mice. (A)** EF distribution to the hippocampus simulated by the FEM. **(B)** The DG region of the hippocampus was assessed for microglia activation modulation. **(C)** The 4-week GES modulated Iba1+ (green) cell activation in DG of 5xFAD mice. The morphological characteristics of microglia were analyzed for the number of Iba1 + cells, cell body size and length, and number of processes from Iba1 + cells. Along with the Iba1 + microglia activation, the reduction of Aβ42 (red) labeling was also detected. DAPI was used as a nuclear counterstain. Scale bars as shown. **(D)** The GES significantly increased the cell count of Iba1 + microglia in 100 and 200 µA groups than that in the sham group. **(E)** The GES significantly decreased the average cell body diameter of Iba1 + microglia in 100 and 200 µA groups than that in the sham group. **(F)** The GES significantly increased the numbers of the average Iba1 + processes in DG of 100 and 200 µA groups than that in the sham group. Data are mean ± SEM from the sham (n = 6 mice), 25 µA (n = 8), 50 µA (n = 8), 100 µA (n = 6), and 200 µA (n = 7) groups. *P < 0.05, **P < 0.01, and *** P < 0.001 were considered significantly different for GES groups vs. sham

**Fig. 4:**
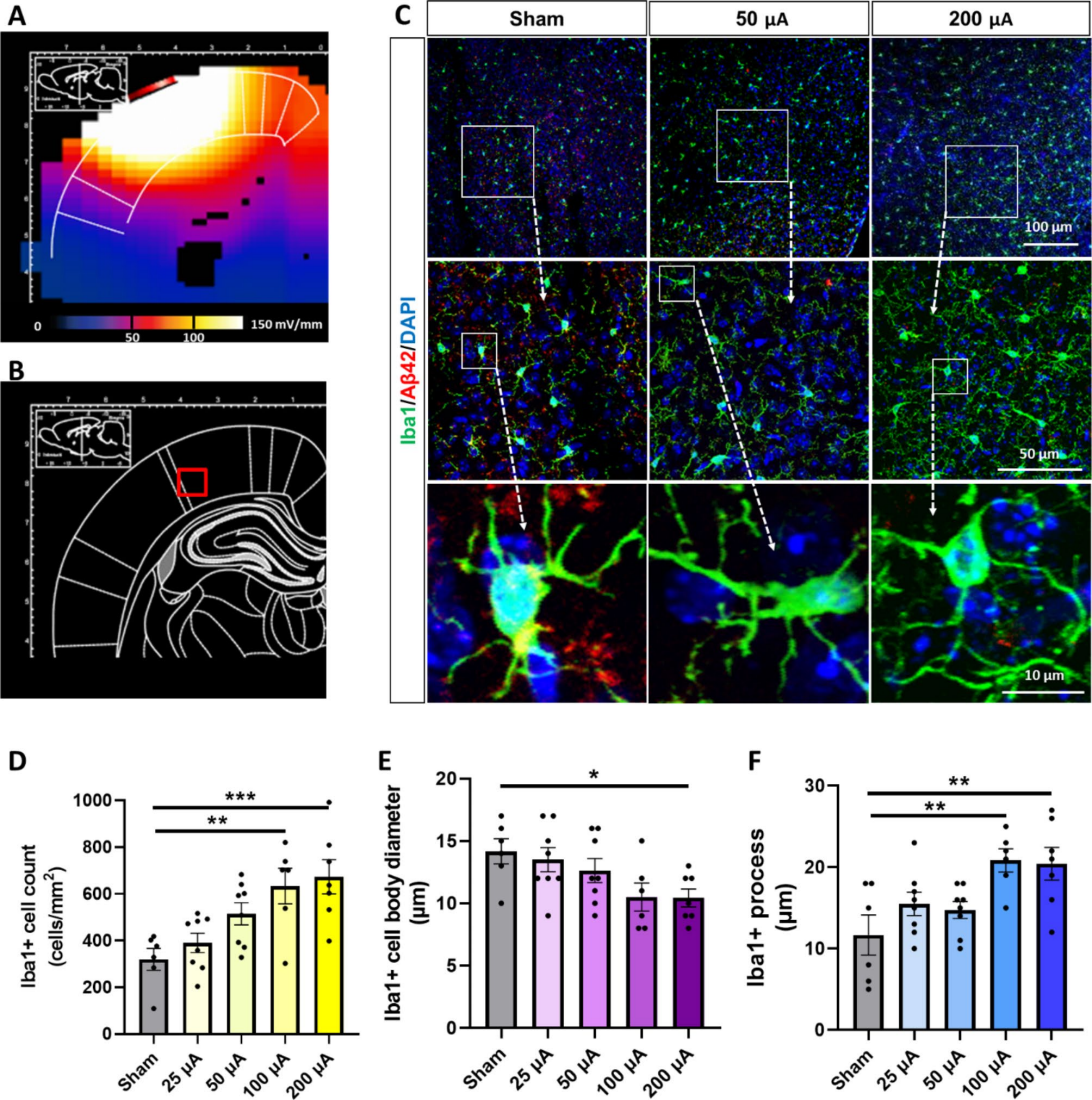
**The GES modulated microglia activation in the cortex of 5xFAD mice. (A)** EF distribution to the hippocampus simulated by the FEM. **(B)** The cortex region was assessed for microglia activation modulation. **(C)** The GES modulated Iba1+ (green) cell activation in the cortex. The morphological characteristics of microglia activation were analyzed for changes in cell body size, extension, and number of cell processes. Along with the Iba1 + microglia activation, the reduction of Aβ42 (red) labeling was also detected. DAPI was used as a nuclear counterstain. Scale bars as shown. **(D)** The GES significantly increased the cell count of Iba1 + microglia in 100 and 200 µA groups than that in the sham group. **(E)** The GES significantly decreased the average cell body diameter of Iba1 + microglia in 50, 100, and 200 µA groups than that in the sham group. **(F)** The GES significantly increased the numbers of the average Iba1 + process in DG of 100 and 200 µA groups than that in the sham group. Data are mean ± SEM from the sham (n = 6 mice), 25 µA (n = 8), 50 µA (n = 8), 100 µA (n = 6), and 200 µA (n = 7) groups. *P < 0.05, **P < 0.01, and *** P < 0.001 were considered significantly different for GES groups vs. sham

As the immune cells in the brain, microglia serve as the main cell type to clear extra pathogens, including Aβ. To address the mechanism of Aβ clearance by GES, we further evaluated the regulation of microglial activation through the Iba1 + cell counts, cell body diameters, and neurite processes [11, 28] in the same brain regions of the hippocampus and cortex. By visual inspection and quantification of the immunofluorescence and microglia morphology, the high-current intensity group demonstrated increased Iba1 + cell counts, decreased cell body sizes, and prolonged neurite processes in both the hippocampus (Fig. 3A C) and cortex (Fig. 4A C). To quantify these observations, Iba1 + cell counts were submitted to ANOVA and exhibited a main effect (F(4, 30) = 11.08, P < 0.0001, for hippocampus; F(4, 30) = 6.634, P = 0.0006, for cortex). In the sham 5xFAD mice, the Iba1 + mean cell counts were detected at 97 ± 11 cells/mm^2^ in the hippocampus (Fig. 3D) and 320 ± 47 cells/mm^2^ in the cortex (Fig. 4D). Compared to the Sham group, the 100 and 200 µA GES treatment groups exhibited a significant increase in the Iba1 + cell counts in both the hippocampus (184 ± 25 cells/mm^2^ in 100 µA GES group, P = 0.01; 207 ± 22 cells/mm^2^ in 200 µA GES group, P = 0.001, Fig. 3D) and the cortex (634 ± 76 cells/mm^2^ in 100 µA GES group, P = 0.006; 672 ± 73 cells/mm^2^ in 200 µA GES group, P = 0.002, Fig. 4D). Although the cell counts were also increased in the 25–50 µA GES treatment groups, there was no significant difference compared to the Sham.

A similar effect of GES was observed on Iba1 + cell body diameters (F(4, 30) = 8.351, P = 0.0001, for hippocampus; F(4, 30) = 2.956, P = 0.0359, for cortex). In the Sham 5xFAD mice, the mean cell body diameter was measured as 17.7 ± 1.3 μm in the hippocampus (Fig. 3E) and 14.2 ± 1.0 μm in the cortex (Fig. 4E). In the 200 µA GES treatment group, the Iba1 + mean cell body diameters in both brain regions were significantly decreased to 8.6 ± 1.2 μm in the hippocampus (P = 0.0002, Fig. 3E) and to 10.4 ± 0.7 μm in the cortex (P = 0.0433 Fig. 4E). Although the mean cell body diameters were also decreased in the 25–100 µA GES treatment group, there was no significant difference compared to Sham.

Again, a similar effect was observed for mean lengths of Iba1 + neurite processes (F(4, 30) = 13.04, P < 0.0001, for hippocampus; F(4, 30) = 5.020, P = 0.0032, for cortex). In the Sham 5xFAD mice, the mean length of neurite processes was measured as 12.5 ± 1.59 μm in the hippocampus (Fig. 3F) and 11.7 ± 2.5 μm in the cortex (Fig. 4F). In the 100 and 200 µA GES treatment groups, the mean length of Iba1 + cell neurite processes in both brain regions was significantly increased in the hippocampus (23.3 ± 2.3 μm in 100 µA GES group, P = 0.003; 25.1 ± 1.9 μm in 200 µA GES group, P = 0.0005, Fig. 3F) and the cortex (20.8 ± 1.4 μm in 100 µA GES group, P = 0.009; 20.4 ± 2.0 μm in 200 µA GES group, P = 0.017, Fig. 4F). Although the process lengths were also increased in the 25–50 µA GES treatment groups, no significant difference was observed compared to the Sham. In summary, the Iba1 + microglial activation in the hippocampus and cortex of 5xFAD mice was regulated significantly by the 40 Hz gamma GES when applied with a ~ 200 µA current intensity, but not with a 25–100 µA current intensity.

### GES enhanced memory function in 5xFAD mice

To assess the effect of GES on the cognitive functions of 5xFAD mice, we performed the MWM assessment [29]. The MWM included two stages of training, and assessment trials to separately measure learning (swimming to find the invisible escaping platform under the water, Training Day 1–4) and memory (on Day 5, the platform was removed, and swimming around the platform area) functions. Learning was assessed and analyzed by 5xFAD mouse swimming efficiency and latency time towards the invisible escape platform in the 4-day training trial of the MWM, before (Week 0) and after (Week 4) the 4-week GES treatment (Fig. 1H**)**. Memory was assessed and analyzed with the time percentage spent at the target quadrant in a 60-second assessment trial of the MWM, before (Week 0) and after the 4-week GES treatment (Fig. 1H**)**.

**Fig. 5:**
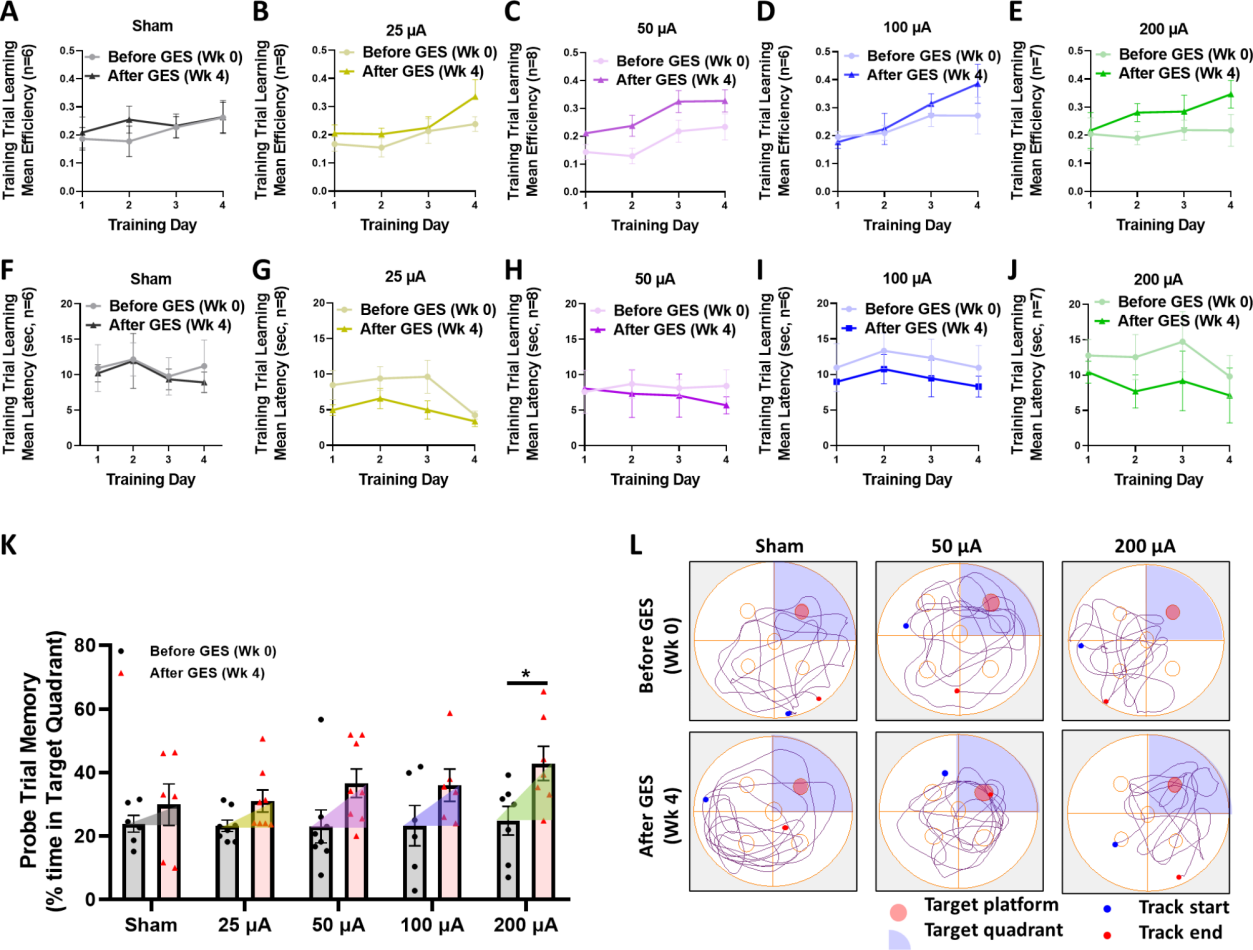
**The GES enhanced learning and memory performance of 5xFAD mice.** The Morris Water Maze test was carried out as detailed in Materials and Methods (Fig. 1) for swimming efficiency and latency to the invisible escape platform in a 4-day training trial before (Wk 0) and after (Wk 4). The memory enhancement was assessed with the time percentage spent at the target quadrant (at which quadrant the platform was removed) in a 60-second probe trial, before and after GES. **A-E.** The swimming efficiency was enhanced in 25–200 µA GES groups. **F-J.** The latency to find the hidden platform was attenuated in 25–200 µA GES groups. **K.** The percentage time in the target quadrant of probe trials showed significantly improved memory in the 200 µA group. **L.** Representative swimming paths before and after the 4-week GES treatment. Data are mean ± SEM from 200 µA group (n = 7 mice), 50 µA group (n = 8), and sham group (n = 6 mice). Data are mean ± SEM from the sham (n = 6 mice), 25 µA (n = 8), 50 µA (n = 8), 100 µA (n = 6), and 200 µA (n = 7) groups. *P < 0.05 was considered as significantly different for after vs. before GES

Assessment of the training data showed improved learning following the 25–200 µA GES treatment, as indicated by swimming efficiency (Fig. 5A and E) and the latency (time in seconds) to the hidden platform (Fig. 5F J). However, the sham 5xFAD mice showed no swimming efficiency improvement or latency decrease. The results suggest that the one-month GES treatment significantly enhanced the learning function of the 5xFAD mice, and the 200 µA GES treatment was more beneficial than those with the lower current intensity.

Assessment of memory, as indexed by the assessment data (Fig. 5K L), showed that the sham 5xFAD mice exhibited no significant improvement in swimming in the Target quadrant from Week 0 to 4 (from 23.8 ± 2.6% to 29.9 ± 6.5%, P = 0.4069). On the other hand, swimming time in the target quadrant improved from Before (Week 0) to After (Week 4) GES in the 200 µA GES treated 5xFAD mice (from 24.8 ± 3.7% at Week 0 to 42.8 ± 5.4% at Week 4, P = 0.0396, Fig. 5K L). However, there was no significance detected in the 25, 50, and 100 µA GES groups. The results demonstrated significantly improved memory performance in the 200 µA GES treatment groups of 5xFAD mice.

## Discussion

In this study, we assessed the effects of GES on biomarkers and behavioral performance measures affected by AD pathology. Compared to a sham control group, one month of GES treatment resulted in lower Aβ42 load in the hippocampus and cortex as well as improved learning and memory performance. Interestingly, these effects were most pronounced in the group that received high-current GES at 100–200 µA. Moreover, the 100–200 µA GES treatment group also exhibited enhanced microglia characteristics, including increased cell counts, smaller cell-body diameters, and longer cell processes. Together, these results highlight the utility of GES as a potential therapeutic intervention to slow or remediate AD pathology.

Electrical activities of the brain that oscillate at 30–100 Hz are grouped as Gamma waves, which have been demonstrated to be involved in neurocircuit function, behavior, and memory [13, 30]. Importantly, multi-center research provides strong evidence suggesting that patients with Alzheimer’s Dementia have decreased Gamma activities [31, 32]. Similarly, in experimental animal models, 5XFAD mice have reduced measures in gamma activities [11]. It is therefore interesting that applying stimulation at this deficient frequency (gamma) can have a positive effect on AD pathology and symptoms.

Various types of brain stimulation to induce gamma oscillations have been investigated for their effects on brain pathology, learning, and memory functions. Those include magnetic, sensory, and optogenetic forms of gamma stimulation. Exciting results from those elegant experiments in animal models of AD have demonstrated improved memory, lowered tau and Aβ load, increased hippocampal long-term potentiation and vascular dilation, and can change activation responses in microglia and astrocytes [10–14].

In contrast, a recent study using multisite silicon probe recording in the cortex and hippocampus found that 40 Hz optical flicker simulation did not induce native gamma oscillations in these regions. In addition, they found no reliable changes in Aβ plaque levels or microglial activation by either immunohistochemistry or in vivo two-photon imaging following the flicker stimulation [33]. The results suggest that optical flicker stimulation may not be a very consistent mechanism nor a method for modulating effectively the neuro-network activity and AD brain pathology.

Whereas, electrical stimulation (intracranial or intra-brain) can directly deliver the 40 Hz electrical signal to neural networks without the involvement of visual or audio sensory mechanisms. Electrical stimulation perhaps can minimize sensory adaptation and avoidance behavior of the animals. Importantly, one of our previous intra-brain electric stimulation studies to guide the migration of neural stem cells in vivo yielded a significant increase in gamma oscillation, but not the theta and beta (please see Fig. S5, panel E. in [34]. That particular result was NOT an intended goal of the original study [34], thus electric stimulation perhaps could be regarded as a more consistent approach to entrain gamma waves. The potential differences between sensory entrainment and direct electrical entrainment will be a future research topic for rescuing the gamma oscillation in various network models considering the conflicting results from sensory inputs [10–14, 33].

Here, we extend these results to the electrical domain to show that GES can lower Aβ42, enhance microglia characteristics, and improve learning and memory. These results converge with our previous report that GES can facilitate neurogenesis [21]. Thus, GES appears to exhibit similar efficacy as the other forms of gamma stimulation. Moreover, these results provide key mechanistic insights as to why GES has exhibited initial efficacy in human trials [17–20].

We also provided novel evidence that GES is intensity-dependent, such that a higher electrical current yielded more pronounced effects. This finding is a critical first step toward understanding the dose-response relationship of this potential therapeutic. The question remains as to what intensity, and for what duration, is optimal for therapeutic use – and how that dose-response curve may differ across the different gamma stimulation techniques.

Precision electrical stimulation of brain structures can be achieved through stereotactic implantation of fine electrodes, which have been well-developed for humans and experimental animals [34, 35]. Stereotaxic brain electrode implantation has been successfully used for various diseases [35–43]. However, complications do happen despite significantly expensive and surgically-demanding procedures [44, 45]. On the other hand, non-invasive stimulation from electrodes on the scalp suffers from poor targeting, and uncertainty of where electric currents would flow and how electric fields may build are not well studied and remain unclear. It is estimated that over 75% of the electrical current from scalp electrodes is attenuated by soft tissue and the skull [46, 47]. Here, using an MRI image-based technique, we were able to simulate the plausibility of using electrodes implanted in the skull and epidural (non-invasive to the brain) to stimulate intracerebral structures. We used a finite element method (FEM) to estimate the distribution of current and applied electric field in a three-dimensional brain model [21–23, 46, 48, 49]. Multiple simulations of the current/field were calculated using electrodes placed at different positions in order to identify electrode positions that would maximally stimulate the cortex and hippocampus. This approach, therefore, permitted an optimal solution for electrode positioning and enabled the targeting of specific structures for stimulation and subsequent tissue analysis via ELISA and immunofluorescence microscopy. GES delivered in such a way indeed has significant effects on brain pathology and animal behaviors, with minimal invasiveness. Indeed, observation of the animal behaviors, and anatomy, and through the histological examination of the brain did not show any detrimental effects from the epidural stimulation procedure.

Microglial activation provides the first line of defense in amyloid plaque clearance whenever injury or disease occurs, including in AD [50–54]. In the early stages of AD, microglia play a beneficial role in reducing Aβ accumulation [55, 56] and can facilitate learning and memory by promoting learning-related synapse formation [57], which helps delay the progression of AD. However, in later stages of AD, persistent microglial activation by amyloid plaques results in a shift from neuroprotective to neurotoxic microglial phenotypes that produce pro-inflammatory cytokines, which in turn can increase Aβ production, neural damage, and are related to tau-mediated neurodegeneration [58, 59]. A recent study reported microglial activation and clustering around amyloid plaques by visual gamma stimulation in Alzheimer’s model mouse brain [11]. Indeed, recent studies have reported microglial activation clustering around amyloid plaques as well as improved memory by auditory and visual gamma stimulation in an Alzheimer’s model mouse brain [10, 11]. This supports the notion that microglial activation may underlie known GES effects on Aβ load and memory performance. Yet, additional research is needed to elucidate the effect of GES on microglial activation as a potential mechanism of Aβ load reduction and improved memory in the AD brain. Moreover, it remains unclear why such a narrow frequency (40 Hz) exhibits efficacy, but not harmonic (80 Hz) or subharmonic (20 Hz) frequencies [11] and whether such frequency specificity would be observed across the different forms of gamma stimulation. There are indeed many critical biological processes, including cell migration, cell proliferation, and cell differentiation that are associated with oscillatory cell signaling [60–66]. Therefore, future research will need to address the mechanisms that underlie frequency specificity. There is a possibility that the electrical stimulation used in our experiments could affect the motor cortex, which might in turn result in more efficient swimming behavior after GES. This can not be excluded nor confirmed in our study. The memory effects are at least independent of the effects on the motor cortex. We are developing more localized stimulation strategies, which will help to determine the effects of stimulation on more specific brain regions.

## Conclusion

In this study, we provided a FEM modeling approach to estimate to which brain parts intracranial electrical stimulation are likely to deliver the electric signal. Importantly, it demonstrated the feasibility of targeting the cortex and hippocampus. The results demonstrated the decreased Aβ load and regulated microglia activation in the hippocampus and cortex of the 5xFAD mice by GES with a sinusoidal alternating current at 40 Hz. With the four-week GES trial, the learning and memory performance was significantly improved in the 5xFAD mice. Furthermore, the GES-induced effects were identified in a current intensity-dependency manner. As similar approaches are under trials in humans to facilitate memory function, our research, therefore, will have direct clinical relevance and significance, which currently affects ~ 5.5 million Americans and many more worldwide.

## Data Availability

The datasets used and/or analyzed during the current study are available from the corresponding authors upon reasonable request.

## Abbreviations

AD: Alzheimer’s disease
5xFAD: The five-familial Alzheimer’s Disease transgenic mouse
EF: electric field
GES: Gamma electrical stimulation
FEM: finite element method
CSF: cerebral spinal fluid
AP: Anterior-Posterior
ML: Medial-Lateral
MWM: Morris Water Maze
PFA: paraformaldehyde
PBS: phosphate-buffered saline
DAPI: 4′,6-diamidino-2-phenylindole
